# The Co-development and Feasibility-Testing of an Innovative Digital Animation Intervention (DAISI) to Reduce the Risk of Maternal Sepsis in the Postnatal Period

**DOI:** 10.1007/s10995-020-02932-4

**Published:** 2020-04-30

**Authors:** Melanie Haith-Cooper, Tomasina Stacey, Fran Bailey, Sarah Broadhead-Croft

**Affiliations:** 1 University of Bradford, Bradford, UK; 2 University of Huddersfield, Huddersfield, UK; 3 National Childbirth Trust, London, UK; 4 Airedale NHS Foundation Trust, Keighley, UK

**Keywords:** Co-design, Maternal sepsis, Postnatal hospital discharge, Behaviour change, Digital animation, Feasibility

## Abstract

**Introduction:**

Sepsis is one of the most common causes of mortality in postnatal women globally and many other women who develop sepsis are left with severe morbidity. Women’s knowledge of postnatal sepsis and how it can be prevented by simple changes to behaviour is lacking.

**Methods:**

This paper describes the co-development and feasibility testing of a digital animation intervention called DAISI (digital animation in service improvement). This DAISI is designed to enhance postnatal women’s awareness of sepsis and how to reduce their risk of developing the condition. We co-designed the digital animation over a six-month period underpinned by theory, best evidence and key stakeholders, translated it into Urdu then assessed its use, firstly in a focus group with women from different Black, Asian and Minority Ethnic (BAME) groups and secondly with 15 clinical midwives and 15 women (including BAME women). Following exposure to the intervention, midwives completed a questionnaire developed from the COM-B behaviour change model and women participated in individual and focus group interviews using similar questions.

**Results:**

The animation was considered acceptable, culturally sensitive and simple to implement and follow.

**Discussion:**

DAISI appears to be an innovative solution for use in maternity care to address difficulties with the postnatal hospital discharge process. We could find no evidence of digital animation being used in this context and recommend a study to test it in practice prior to adopting its use more widely. If effective, the DAISI principle could be used in other maternity contexts and other areas of the NHS to communicate health promotion information.

## Significance

Using digital animation to impart important health messages to patients has been effective in different health service contexts. No evidence could be found of its use at the postnatal hospital discharge, which currently in the NHS can be inadequate and rushed. This paper demonstrates that a co-designed sepsis prevention digital animation, viewed by postnatal women prior to and following hospital discharge is acceptable, culturally sensitive and simple to implement and follow and therefore could be effective in changing women’s behaviour. In addition, ensuring the digital animation is culturally and linguistically sensitive may make it acceptable for use by women from BAME backgrounds, who are more likely to become ill with sepsis following childbirth.

## Introduction

The postnatal hospital discharge is an important transition in maternity care, with women requiring a plethora of information to avoid preventable morbidity and mortality (NICE 2003) Evidence has found that the postnatal hospital discharge process can be inadequate, rushed and inconsistent, with insufficient information provided to women (Haith-Cooper et al. 2018; Suplee et al. 2017) Information quality varies and is usually provided in the form of paper or links to electronic leaflets which are time consuming to read and problematic for women with language barriers or poor health literacy. Ten percent of pregnant women in the UK speak limited English and they are at an increased risk of poor maternal outcomes (NICE 2010). In addition, evidence suggests that trained interpreters are not consistently used in maternity services (Haith-Cooper 2014; Phillimore 2016).

Globally, there are over 800 preventable deaths every day related to pregnancy and childbirth (WHO 2019). Sepsis (most commonly postpartum) is one of the leading causes of maternal death worldwide and accounts for over 10% of deaths (Bonet et al. 2017; Say et al. 2019). For every death, thousands of women experience severe morbidity. More than 60 women each year die due to childbirth, half of which occur in the postnatal period (Knight et al. 2017). In the UK, more than 60 women die each year due to childbirth, half of these deaths occur in the postnatal period (Knight et al. 2017) with higher rates in women from Black, Asian and Minority Ethnic (BAME) groups who may speak limited English (Acosta et al. 2014; Mohamed-Ahmed et al. 2015). Sepsis is the result of the body’s over-reaction to an infection and key health messages such as keeping wounds clean and dry, regular handwashing can help prevent infections and the development of sepsis and responding to the symptoms of sepsis in a timely manner can improve outcomes (NHS 2020).

The length of the postnatal hospital stay has reduced with the UK, which now has one of the shortest lengths of postnatal stays in the world (Beake et al. 2010). At the same time, readmission rates with postnatal maternal complications are increasing (NMPA 2017; RCOG 2013). A combination of time pressures, possible language barriers and the need for complex advice to prevent postnatal complications has led to a reduction in the quality of the postnatal discharge process (Haith-Cooper et al. 2018). There is a need to find novel ways to improve the quality of the delivery of this episode of care, ensuring that adequate, accessible and consistent information around conditions such as sepsis is given to all women. Digital animation could be used to provide simple health messages in the woman’s chosen language and between 92–95% of people of childbearing age own a smart phone (Statista 2018). There is increasing evidence that digital animation can be effectively used in health care (Jack et al. 2009), but a systematic literature search of key databases (Medline, CINAHL, PsycINFO, Embase and MIDIRS) using the search terms: *digital animation, animation, cartoon, app* combined with *postnatal, postpartum, perinatal, peripartum*, found no research around its use as part of the postnatal hospital discharge process. Information regarding the prevention of maternal sepsis is an example of health promotion information that should be provided at the postnatal hospital discharge (NHS England 2016). However, discussion around sepsis at discharge is inconsistent, with many women not being familiar with the condition (Haith-Cooper et al. 2018)*.* In this paper, we describe the co-design, development and pilot testing of a digital animation intervention called DAISI (digital animation in service improvement) which focuses on postnatal maternal sepsis. DAISI was developed to enhance postnatal women’s awareness of their risk of developing sepsis, as an example of information required at the postnatal discharge.

## Methods

DAISI was developed over a six-month period, through an iterative co-design process with key stakeholders. This ensured that the emerging animation was informed by the knowledge and background of people who would be subsequently engaging with it (Langley et al. 2018). A skilled facilitator guided discussion of different stakeholders including clinical midwives and women with infants (including women from BAME backgrounds) to ensure all opinions were equally valued. Development was guided by the Medical Research Council framework for complex interventions (Craig et al. 2008) and was underpinned by the best available evidence on preventing sepsis postnatally (Bonet et al. 2017). The principles of the Com-B behaviour change model (Michie et al. 2011) were applied to the stages of the development process by judging whether the evolving animation could influence women’s capability, motivation and opportunity to follow the advice in the animation.

The co-design group formally met on three occasions with e mail and phone contact maintained in between to gain opinions on specific aspects of the animation as they emerged. The initial meeting involved agreeing how the key messages to prevent sepsis could be communicated through DAISI to increase the capability, opportunity and motivation of women to change their behaviour to prevent postnatal sepsis. Designing an avatar that was considered culturally appropriate and representative of a postnatal woman was an important discussion point and how we could maintain dignity whilst pictorially demonstrating and describing intimate procedures such as changing sanitary pads frequently. The developers produced a prototype which was reviewed at the second meeting with suggestions for changes to the design and content to optimise the opportunity for the animation to change behaviour, which were then reviewed and signed off at the third meeting.

**Fig. 1:**
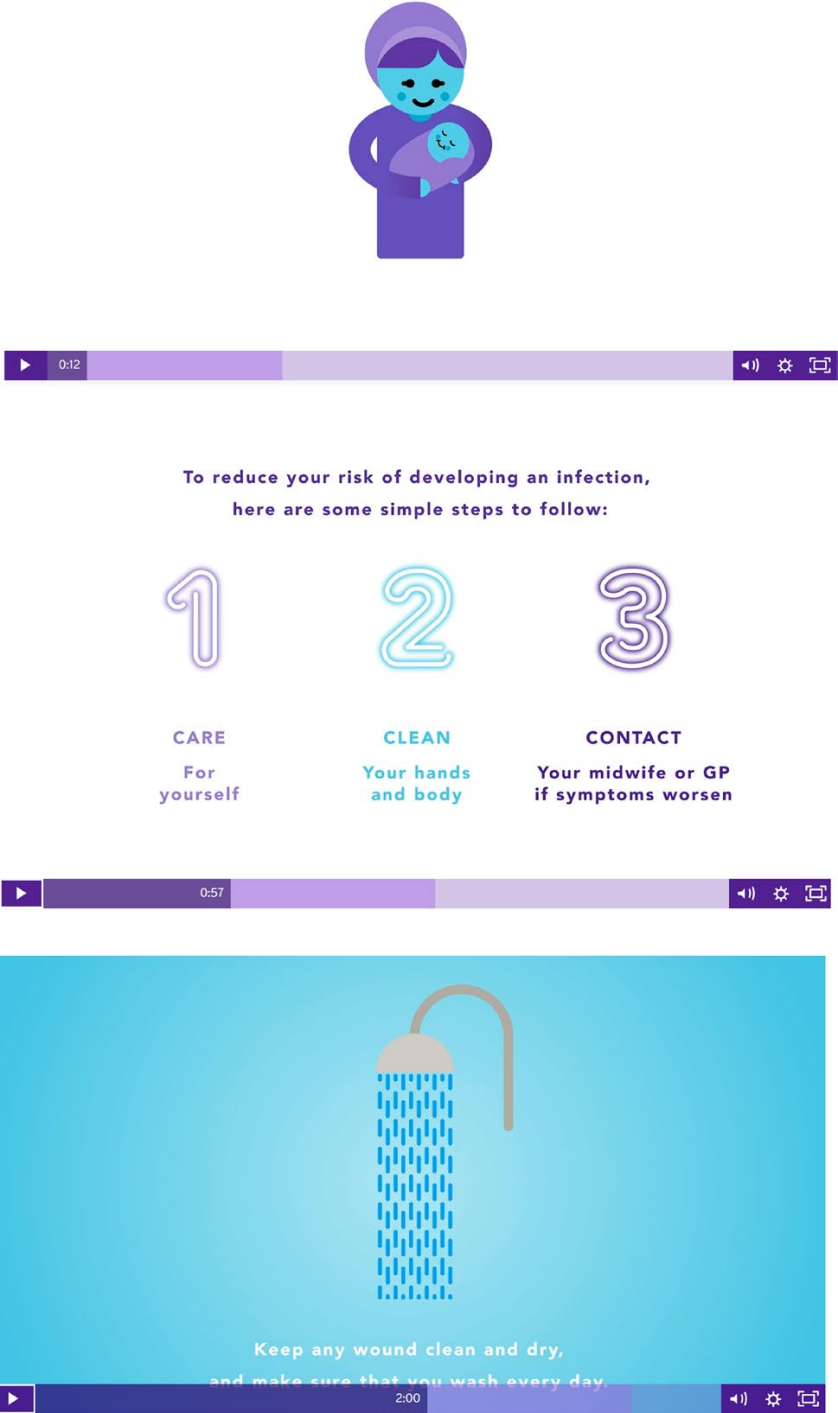
Screenshots from DAISI

The final version of DAISI (Fig. 1) is a 3 min animation comprising of a purple coloured female avatar dressed in clothing covering her hair and body. The animation is divided into three sections each with a key behaviour change message to reduce the risk of developing sepsis; (the three Cs) **c**are for yourself through diet, adequate fluids and rest, keeping **c**lean (personal hygiene) and who to **c**ontact should the woman develop signs and symptoms of sepsis—including timely medical attention if symptoms develop. She demonstrates simple behaviour change to reduce the risk of women developing postnatal sepsis and responding appropriately to signs and symptoms. The animation includes subtitles and a voice over currently available in English and Urdu. Translation into Urdu was undertaken by a bilingual midwife and another bilingual woman checked the content against the English subtitles to accuracy in terms of original meaning and content.

We then undertook a study with women and midwives with the aim of assessing the feasibility and acceptability of using DAISI as a tool to change behaviour and reduce the risk of developing maternal postnatal sepsis. The study was approved by the Chair of the Humanities, Social and Health Sciences Research Ethics Panel at the University of Bradford on 22nd June 2018 ref EC25146 and has therefore been performed in accordance with the ethical standards laid down in the 1964 Declaration of Helsinki and its later amendments. We followed the COREQ criteria for reporting qualitative research.

Purposive sampling was used to recruit 15 women and 15 clinical midwives, a pragmatic decision based on funding and timescale available. In addition, the study aimed to gain insights into the feasibility of implementing DAISI into clinical practice rather than reaching data saturation. Women were included if they had a baby in the last two years, and half of the women represented a BAME group to provide a different cultural perspective. Women were excluded if they had a poor pregnancy outcome or spoke a language for which we could not find an interpreter. Women were approached and recruited through mother and baby groups that are non-Governmental organisations. Midwives were eligible to take part if they currently worked in NHS maternity services. They were approached and recruited by SBC through the personal messaging option on the social media sites Linked in, Facebook and Twitter.

Women and midwives were provided with an information leaflet which was discussed with them by a member of the research team. Audio-recorded verbal informed consent was acquired as some BAME women may have feared providing a signature due to concerns about their immigration status. Implied consent was assumed for the midwives who opted to complete the survey. Anonymity and confidentiality were assured and requested by women being interviewed in groups. All data were anonymised and no data included which could reveal the identity of a participant.

Data were collected from the women using a combination of individual interviews (n = 7) and two group interviews with four women in each. They were all conducted by FB who was familiar to the women. Midwives completed online questionnaires. The questions for both tools (see Table 1) were based on the Com-B behaviour change model (Michie et al. 2011). For the women, the questions were stemmed around whether the user could understand and recall the advice in the animation and if they believed that women would be able to follow the advice in the digital animation and therefore change their behaviour to reduce the risk of developing sepsis. The interview questions were checked by a BAME woman who was a service user. The interviews lasted between 20 min and 1 h and data were audio recorded and transcribed verbatim. Field notes were not used in this context. The questionnaires were based around the same themes with questions related to whether the midwife believed the advice in the digital animation could motivate women to change their behaviour. Midwives were also asked whether they would like to use the digital animation at the postnatal hospital discharge. All participants were asked to recall the key messages from the animation and their perspectives on the social and cultural acceptability of the content.

**Table 1:** Development of the questionnaires and interview schedules using the Com-B behaviour change model (Michie et al. 2011)

Questionnaire for midwives	
Question	Link to Com-B model
What do you think were the key messages from the animation?	Capability in relation to key messages and level of information provided
Do you think these were accurate?	
Do you think they were pitched appropriately for postnatal women?	
Do you think the animation is sensitive to women from different backgrounds? (social and cultural)	Opportunity- ensuring the content does not create a social or cultural barrier
In what ways?	
Do you think the animation would motivate women to follow the advice?	Motivation
In what ways?	
How do you feel about using this animation at the postnatal hospital discharge?	Midwives’ motivation, capability and opportunity
Can you suggest any changes to the animation that would make it better?	
Length of animation	
Use of graphics	
Use of words	

Interview schedule for women	
Question	Link to Com-B model
What were the key messages from the animation?	Capability to recall the key messages
Do you think the animation was sensitive to your background (social and cultural)	Opportunity- ensuring the content does not create a social or cultural barrier
How?	
Do you feel you would want to follow the advice?	Motivation
Why?	
Do you think you would be able to follow the advice on the animation?	Opportunity and capability
If not why not?	
Can you suggest any changes to the animation that would make it better?	
Length of animation	
Use of pictures	
Use of words	

Descriptive statistics were used to analyse the midwives’ questionnaire responses and deductive thematic analysis was used to analyse the qualitative data from the interviews using word documents, to allow the theoretical underpinnings of the Com-B model to drive the development of codes and themes (Attride-Stirling 2001). Themes emerged which related to the three areas of the Com-B model. To ensure credibility of the process, coding and themes were developed by two members of the research team (MC and TS).

## Results

Fifteen women participated in the study. Of these women eight were white British and seven women were from BAME backgrounds (including Pakistani, Syrian and Albanian), of these women one required an interpreter. Fifteen clinical midwives participated, with 1–30 years of clinical experience and representing three different NHS Trusts. After viewing DAISI, all participants could accurately recall the key messages and they all believed it was acceptable for women from different sociocultural backgrounds. They perceived it could be used to change women’s behaviour around preventing sepsis, if presented in the woman’s chosen language. Interestingly, after viewing DAISI, one woman realised that she had sepsis after her first child was born and had not known what was wrong with her at the time.

Most midwives completed the online questionnaire superficially resulting in limited data. Fourteen midwives believed that DAISI could motivate women to follow the advice and reduce their risk of developing sepsis. One midwife was unsure but believed it would at least raise awareness in women. Thirteen of the midwives believed the animation was feasible to use at the postnatal hospital discharge, feeling confident they had the ability to use DAISI as it provided a simple, quick and consistent message that would be easy to implement Two midwives didn’t agree with the timing of its use believing it could be used additionally in the antenatal period and several midwives believed that it would be additionally useful to use with partners, families and teaching midwives.

All the women were positive about DAISI believing that it was acceptable and should be used with postnatal women. From the deductive thematic analysis process, three themes emerged (simplicity and motivation, cultural acceptability and time and capability) which identified potential barriers and facilitators to women having the capability, opportunity or motivation to follow the advice in the animation. The transcribers could not differentiate individual women’s contributions, consequently the findings are classified by interview (Int) and number and identifies BAME women within the interview.

### Simplicity and Motivation

Most women felt that they would feel motivated to follow the advice provided in DAISI due to the design; the simplicity of the 3 Cs and being able to remember the messages easily. They believed the messages were structured in a way to make the importance of the subject clear.

One woman discussed how DAISI was particularly motivating, being a single parent in a foreign country:

When you have a baby especially when you are in(on) your own, it’s very difficult everything because you have to manage everything yourself….and when you see (DAISI) it’s good to remind that to be careful. (BAME woman Int7).

Some women felt motivated as DAISI helped to increase their knowledge of sepsis and what they needed to do to reduce their risk of developing the condition. DAISI provided new information that they had not been provided at their postnatal hospital discharge:

..I did a few things, well, not all of the things they mentioned …changing your pad every time you go to toilet, I didn't do that. So if I knew that, that would be, you know, better for me (BAME woman Int7).

Some women discussed how they would be motivated to share their new knowledge with friends and family who they thought would benefit from the advice.

Women discussed how DAISI would motivate them to seek advice quicker if they developed symptoms of sepsis and how DAISI would motivate them to get checked out:

if you are not OK, so how do you look after your children? So you must go to the GP and consult your midwife and doctor as well (BAME woman Int4).

Most of the women felt the availability of DAISI in different languages would increase the capability for women with low levels of English to follow the advice and recommended that we increase the number of languages available.

### Cultural Acceptability

Women believed that the design of DAISI was culturally sensitive so would not cause offence when viewed by women from different cultural backgrounds. Specifically, women felt that the avatar being purple, not resembling women from a particular ethnic background and not showing detail of a style of house in the background increased acceptability.

Yeah, yeah, it was, it didn’t favour either white people or any different races or anything, it was very multicultural, you wouldn’t think it was specifically directed towards a certain race or anything (BAME woman int6).

Modesty was also an important issue for some women:

…your private parts…they made..carefully so that it doesn't hurt anybody's culture or anybody's feelings…anybody can watch that…I feel confident to watch that and I can watch that with the men as well, I don't mind, yeah, because it doesn't harm you anyway, it doesn't like disrespecting, you know, a woman. So I think it's a good cartoon to watch. (BAME woman, Int2).

Cultural beliefs around the caring role of women appeared to influence some women’s belief about their opportunity to follow the advice in DAISI. In some cultures, the caring role was a barrier:

…some Asian mums don’t because they say, “My house is messy, my house is messy, what I do, I clean that thingy, that thingy,” and forgot about our health (BAME woman Int4).

However, in other cultures, the norm for postnatal women is to rest for 40 days following childbirth was considered a facilitator to following the advice:

..just from the bed, got to toilet and just eating or just walk around, no baby (to) look after, just feed and lie down, my mum everything doing… (BAME woman Int3).

Some women also believed that as there was no mention in the animation of partners or families, it would not offend women living in different family structures:

So, it doesn’t matter if you were single or in a relationship and no matter what kind of relationship, if it’s male–female or female-female or male-male, it doesn’t matter. (woman Int1).

The social background of the BAME woman was considered to influence the capability to follow the advice in the animation:

..city ladies…mostly do it…but village lady can’t understand, …ladies not go to school or can’t understand what you say or what I am say (BAME woman Int3).

Other women expressed concern about having the opportunity to follow some of the advice due to the conditions in which they were living:

I have been in difficulties and to have a bath …you take your baby with you in your bathroom, bathroom is shared because we live in sharing house (BAME woman Int7).

However, other women didn’t agree with this perspective believing all women can follow the advice:

it’s not difficult to follow this kind of advice, anybody can. Absolutely, this is to do with the cleanliness, hygiene, of course one has to be following this kind of procedure, yeah (BAME woman Int8).

### Time and Capability

Women felt that the length of time it took to view the animation would encourage women to view it again. However, a lack of time due to the demands of a new baby was considered a potential barrier to women being able to follow the advice in the animation. An example was the urgent nature of feeding the baby and the recommendation to wash hands before and after the procedure:

…sometimes you’re just desperate to feed your baby or change your baby and you just have to quickly do it. Especially if you’re not in the house, you know, if you’re out it’s not always easy to get to a sink to wash your hands…I always try to have hand sanitiser with me just to be on the safe side, but again, sometimes I don’t even manage to get that out of my bag. (woman Int1).

A Lack of time also related to the woman ‘caring for herself’ aspect of the animation and the time constraints affecting capability to follow this advice:

..sleeping when the baby sleeps …You have to have a shower, don’t you, when the baby sleeps? Or feed yourself….(woman Int1).

Some women discussed the physical exhaustion associated with caring for a new baby and the negative impact on following the advice from the animation:

Yeah, so even if you had that advice in the back of your mind I don’t know that you are thinking that straight, if you know what I mean? You’re just trying to get through the first few days, aren’t you? Without trying to think about am I washing my hands? Have I had a shower every day? Right? Have I eaten something healthy? Because you’re just so exhausted (woman Int1).

Despite these barriers, the women believed the purpose of DAISI was to provide information and even if women found time constraints, it should still be used in practice:

I think it was all quite sensible advice as well, I don’t think there was anything that felt like it …would be difficult to think to remember to do it. It’s just, like I say, having the time sometimes to wash your hands and things. (woman Int1).

## Discussion

Overall, participants were positive that the intervention could be an acceptable tool to facilitate women in the postnatal period to change their behaviour to reduce their risk of developing sepsis. Most midwives felt that they would like to use DAISI within their current postnatal practice, and the women believed it should be used postnatally.

The women identified several barriers and facilitators to women following the advice in DAISI. The facilitators related to the simplicity of DAISI, the information provided to increase knowledge, its cultural acceptability and the potential for it to be available in different languages. Women believed this simplicity would increase motivation and also the opportunity for more women to follow the advice. There were barriers identified which influenced the capability of women to follow the advice. These barriers linked to a lack of time and physical exhaustion due to having a new baby and also different cultural roles of women. However, cultural practices could also be a facilitator with female family members to support the new mother. These barriers could not be addressed through adjusting the digital animation, they relate more to women’s roles in society and wider structural barriers to women caring for themselves with a new baby.

Although we could find no previous research using digital animation in the context of the postnatal hospital discharge, the potential highlighted from our findings support previous studies around the use of mobile applications to provide advice on baby care at the postnatal hospital discharge process (Danbjørg et al. 2014; Danbjørg et al. 2015). Mobile applications increased parents’ perceptions of the quality of the information provided and also their confidence in caring for their new baby. The potential from our findings also support previous studies which have successfully implemented digital animation in different areas of health care including vaccination, medication and colon cancer advice and information (Schnellinger et al. 2010). Like our findings, other studies have found that digital animation to be an acceptable form of providing health messages (Meppelink et al. 2015; Schnellinger et al. 2010) with one study leading to an increased likelihood of sustained positive behaviour change (Schnellinger et al. 2010). The largest study to date (Project RED) which used personalised digital animation found a 30% decrease in hospital re admissions with complications post-operatively (Jack et al. 2009). Our findings appear to have the potential for DAISI to influence women changing their behaviour to reduce their risk of developing maternal sepsis.

A perceived facilitator to following the advice in DAISI was the cultural sensitivity of the animation and the potential availability in different languages. We believe cultural sensitivity is an important finding and one that we could not find any previous research to support. Persistent language barriers in midwifery care increase the risk of preventable morbidity and mortality in BAME women and is a threat to patient (CMACE 2011; NICE 2010)The use of trained interpreters to address language barriers in maternity care is inconsistent (Haith-Cooper 2014) and our findings suggest DAISI in the woman’s chosen language could be used to overcome language barriers, understand key health messages and reduce BAME women’s risk of maternal illness. In addition, our findings suggest that the selection of the colour palate, neutral avatar, discreet clothing and culturally neutral background could ensure women are not offended by the content and therefore are more likely to view the animation.

The limitations of the study include superficial data from the midwives responses and a potential selection bias due to a small, local sample of self-selecting women with infants obtained from non-Governmental organisations and midwives who could be accessed via social media. However, women were ethnically diverse and the midwives worked in three different NHS Trusts suggesting different backgrounds and perspectives were included. This diversity increases the likelihood that these findings being transferable to other women and other maternity sites.

## Conclusions

In this paper, we discussed the co-design, co-development and feasibility testing of the DAISI intervention, a short animation on a SMART phone designed to provide information and change women’s behaviour to reduce their risks of developing postnatal sepsis. The three stages of the animation- care, clean and contact were assessed by participants in relation to the Com-B model, the impact on a woman’s motivation, ability and opportunity to change her behaviour in this context.

As DAISI was received positively by all study participants, we would suggest that further testing is required to assess the impact of the intervention when used in clinical practice. If found to be effective in changing women’s health behaviour, the use of DAISI could be expanded to provide key health messages about other aspects of the postnatal hospital discharge, other aspects of maternity care and also more widely used in health services. It could also be used in teaching midwifery students and parents in antenatal classes.
